# Treinamento com Exercício Físico e Doença de Chagas: Função Potencial dos MicroRNAs

**DOI:** 10.36660/abc.20200330

**Published:** 2021-07-15

**Authors:** Alex Cleber Improta-Caria, Roque Aras

**Affiliations:** 1 Programa de Pós-Graduação em Medicina e SaúdeFaculdade de MedicinaUniversidade Federal da BahiaSalvadorBABrasilPrograma de Pós-Graduação em Medicina e Saúde, Faculdade de Medicina, Universidade Federal da Bahia, Salvador, BA - Brasil; 2 Departamento de Educação Física em Cardiologia do Estado da BahiaSociedade Brasileira de CardiologiaSalvadorBABrasilDepartamento de Educação Física em Cardiologia do Estado da Bahia, Sociedade Brasileira de Cardiologia,Salvador, BA - Brasil

**Keywords:** Exercício Físico, Doença de Chagas, MicroRNAs

## Abstract

A doença de Chagas (DC) é causada pelo *Trypanosoma Cruzi*. Esse parasita pode infectar vários órgãos do corpo humano, especialmente o coração, causando inflamação, fibrose, arritmias e remodelação cardíaca, e promovendo a cardiomiopatia chagásica crônica (CCC) no longo prazo. Entretanto, poucas evidências científicas elucidaram os mecanismos moleculares que regulam os processos fisiopatológicos nessa doença. Os microRNAs (miRNAs) são reguladores de expressão gênica pós-transcricional que modulam a sinalização celular, participando de mecanismos fisiopatológicos da DC, mas o entendimento dos miRNAs nessa doença é limitado. Por outro lado, há muitas evidências científicas demonstrando que o treinamento com exercício físico (TEF) modula a expressão de miRNAs, modificando a sinalização celular em indivíduos saudáveis. Alguns estudos também demonstram que o TEF traz benefícios para indivíduos com DC, porém esses não avaliaram as expressões de miRNA. Dessa forma, não há evidências demonstrando o papel do TEF na expressão dos miRNAs na DC. Portanto, essa revisão teve o objetivo de identificar os miRNAs expressos na DC que poderiam ser modificados pelo TEF.

## Introdução

A doença de Chagas (DC) é uma doença complexa causada pela infecção por *Trypanosoma Cruzi *(*T. Cruzi*), um parasita protozoário flagelado, no nível intracelular.^1^ Na fase aguda, a infecção por *T. Cruzi* gera grande inflamação dos tecidos e há uma resposta inicial do sistema imune inato na tentativa de combater a parasitemia.^2^

Entretanto, a infecção persiste e o sistema imune adaptativo ativa tanto os linfócitos T, como também as células T citotóxicas e auxiliares, que produzem citocinas, tais como o interferon gama (IFN-γ), que podem, por sua vez, levar à morte de parasitas intracelulares ao induzir um aumento nas espécies reativas do oxigênio e nitrogênio, que são microbicidas. Essa infecção também aumenta a expressão do fator de necrose tumoral (TNF-α), bem como anticorpos específicos para combater o *T. Cruzi*, que controlam o parasitismo, com o estabelecimento de uma infecção de baixo grau.^3^

Ainda na fase aguda da doença, há um aumento na expressão do peptídeo vasoativo endotelina-1 (ET-1) e da cardiotrofina-1 (CT-1), ambos induzindo a hipertrofia cardíaca, bem como um aumento na expressão da interleucina-1 beta (IL-1β), induzindo uma resposta inflamatória e pró-hipertrófica do miocárdio, o que pode iniciar a hipertrofia até mesmo nesse estágio.^4,5^

Com o passar dos anos, a parasitemia diminui; entretanto, os antígenos parasíticos persistem, gerando um infiltrado inflamatório difuso e miocardite, com a presença de linfócitos T CD4+ e CD8+ e macrófagos que continuam a expressar TNF-α e IFN-γ.^3^ O IFN-γ tem a função essencial de controlar e combater parasitas, mas também contribui para a patogênese cardíaca, uma vez que lesiona o miocárdio por vários mecanismos moleculares que geram a disfunção miocárdica.^6^

Portanto, a doença evolui e passa para a fase crônica, que pode ser subdividida em duas formas: indeterminada e sintomática. Na forma indeterminada, os indivíduos podem passar anos sem manifestar nenhum tipo de sintoma mais sério, já que existe um equilíbrio entre a parasitemia e o sistema imune do hospedeiro. Entretanto, cerca de 30% desses pacientes desenvolvem uma forma sintomática ou determinada, que pode desencadear disfunções em vários órgãos, incluindo o coração, desenvolvendo a cardiomiopatia chagásica crônica (CCC) associada à miocardite e a fibrose miofibrilar cardíaca, e, dessa forma, reduzindo a condutividade elétrica cardíaca, levando à remodelação miocárdica.^7^

A CCC gera inflamação do tecido cardíaco, causando miocardite focal ou difusa, hipertrofia ou dilatação do ventrículo esquerdo, e morte progressiva de alguns cardiomiócitos, necrose e depósito de colágeno,^8^ aumentando, assim, o tecido fibrótico, levando à redução de sua capacidade de contração. Esse resultado é geralmente associada a arritmias e insuficiência cardíaca,^9^ mas é possível que os microRNAs (miRNAs) participem desses mecanismos. Em geral, os mecanismos moleculares que regulam esses processos não são bem entendidos.

MiRNAs são pequenos RNAs com comprimento de apenas 18 a 25 nucleotídeos,^10^ proteínas não codificantes, e reguladores da expressão gênica pós-transcricional com a função de inibir ou degradar seus genes alvo.^11,12^ Já se demonstrou que vários tipos de treinamento com exercício físico (TEF) modulam a expressão dos miRNAs.^13^ Entretanto, há poucos artigos na literatura que tenham analisado os efeitos do TEF na expressão dos miRNAs na DC. Portanto, o objetivo desta revisão de literatura foi analisar os miRNAs expressos na DC e comparar esse resultado aos miRNAs expressos durante ou depois do TEF.

### Doença de Chagas e miRNAs

Poucos estudos na literatura analisaram o perfil de expressão de miRNAs na DC, seja na fase aguda ou na crônica, bem como a sinalização celular que é regulada pelos miRNAs nessa doença negligenciada. Portanto, este trabalho incluiu todos os estudos que avaliaram o padrão de expressão dos miRNAs na DC (Tabela 1).

**Tabela 1:** – MicroRNAs na doença de Chagas

MicroRNAs	Fonte	Achados	Referência
↓ miR-1, miR-133a, miR-133b, miR-208a, miR-208b	Amostras cardíacas	Associação a distúrbios do tecido conjuntivo e fibrose	16
↑ miR-208b	Amostras plasmáticas	Associação à disfunção cardiovascular e hipertrofia miocárdica	17
↑ miR-20, miR-20b, miR-21, miR-142, miR-146a, miR-146b, miR-155, miR-182, miR-203, miR-222 ↓ miR-139, miR-145, miR-149, miR-322, miR-503,	Amostras cardíacas	Associação com o intervalo QT (QTc) corrigido pela frequência cardíaca. Despolarização e repolarização ventricular.	14
↑ miR-19a, miR-21, miR-29b, miR-30a, miR-199b	Amostras cardíacas e modelo celular	Associação à fibrose e remodelação cardíaca	18
↑ miR-16, miR-26b, miR-190b, miR-3586, let-7f-2 ↓ miR-190b	Células H9c2, infectadas com *T. Cruzi*	Associação com crescimento celular, hipertrofia e sobrevivência celular	19

#### Doença de Chagas (fase aguda) e miRNAs

Durante a fase aguda da DC, os pesquisadores avaliaram a expressão de miRNAs aos 15, 30 e 45 dias após a infecção, e identificaram que os miRNAs se expressaram diferentemente durante a parasitemia e que mudanças no intervalo QT sofreram regulação ascendente: miR-20, miR-20b, miR-21, miR-142, miR-146a, miR-146b, miR-155, miR-182, miR-203, miR-222, e descendente: miR-139, miR-145, miR-149, miR-322, miR-503.^14^

Outro estudo realizou uma análise *in silico* para identificar a expressão diferencial de miRNAs e seus genes alvo em vários processos biológicos durante a fase aguda da infecção pelo* T. Cruzi*, demonstrando que alguns podem estar associados ao processo patológico, tais como os miRNAs miR-238-3p, miR-149-5p, miR-143-3p, miR-145-5p e miR-486-5p. Outros miRNAs podem estar associados à imunidade e função cardiovascular, por exemplo: miR-10a-5p, miR-16-5p, miR-30c-5p, miR-34a-5p, miR-138-5p, miR-146a-5p, miR-149, miR-191-5p, miR-204-5p, miR-320b e miR-653-3p, bem como miRNAs relacionados ao processo de fibrose de tecidos: miR-34a-5p, miR-142-3p, miR-200b-3p e 203a-3p.^15^

#### Doença de Chagas (fase crônica) e miRNAs

A expressão dos miRNAs do tecido cardíaco dos pacientes com CCC após o transplante cardíaco foi analisada e comparada à expressão de miRNAs do tecido cardíaco de doadores saudáveis. De todos os miRNAs analisados, cinco tiveram sua expressão reduzida (miR-1, miR-133a, miR-133b, miR-208a e miR-208b) em pacientes com CCC em comparação ao grupo de controle.^16^ Em contraste, o miR-208a circulante em uma amostra de plasma foi superexpresso em pacientes com DC. Entretanto, eles foram indeterminados na fase crônica.^17^

A superexpressão de miR-19a, miR-21 e miR-29b já foi descrita em pacientes com CCC em comparação a indivíduos saudáveis. Inclusive, na análise histológica do tecido cardíaco de pacientes no estágio final da CCC, identificou-se que, além desses miRNAs mencionados acima, o miR-30a e o miR-199b também são superexpressos na DC.^18^

Esses estudos demonstram que muitos miRNAs participam de vários processos na DC, tanto na fase aguda quanto na crônica. Entretanto, são necessários mais estudos para elucidar o papel desses miRNAs e a sinalização celular que estão regulando na DC, incluindo a importância de terapias e tratamentos que podem modular o padrão de expressão apresentado na doença.

## Doença de Chagas e treinamento com exercício físico: miRNAs como possíveis moduladores

Vários tipos de TEF foram descritos como moduladores da expressão de miRNAs,^13^ em estudos experimentais e clínicos, tais como TEF de natação,^20^ maratona,^21^ corrida em esteira^22^ e treinamento de resistência (TR)^23^ (Tabela 2).

**Tabela 2:** – MicroRNAs em Treinamento com exercício físico (estudos pré-clínicos e clínicos)

MicroRNAs	Alvo	Fonte	Tipos de exercícios	Referência
**Modelos experimentais *in vivo***				
↑** miR-27a, miR-155** ↓** miR-143**	ACE, AT1R	Amostras cardíacas	Ratos Wistar-Kyoto Treinamento físico na esteira	39
↑ **miR-17-3p**	TIMP-3 PTEN	Amostras cardíacas	Ratos C57Bl/6 Modelo de treinamento de nado em rampa Treinamento em roda voluntário	40
↑ miR-222	HIPK1	Amostras cardíacas	Modelo de nado em rampa Treinamento em roda voluntário	41
↑ miR-19b, miR-30e, miR-133b, miR-208a ↓ miR-99b, miR-100, miR-191a, miR-22, miR-181a	IGF-1 PI3K/AKT/mTOR MAPK	Amostras cardíacas Plasma	Ratos Wistar albinos Treinamento de natação	42
↑ miR-29a, miR-101a	TG-β fos COL1A1	Amostras cardíacas	Exercício de corrida intermitente	43
↑ miR-27a, miR-27b ↓ miR-143	ACE ACE2	Amostras cardíacas	Ratos Wistar Treinamento de natação	44
↑ miR-126	PI3KR2	Amostras cardíacas Plasma	Ratos Zucker Treinamento de natação	26
↓ miR-214	SERCA2A	Amostras cardíacas	Ratos Wistar Treinamento de resistência	23
↑ miR-1 ↓ miR-214	NCX SERCA2A	Amostras cardíacas	Ratos Wistar Treinamento de natação	27
↑ miR-29c ↓ miR-1, miR-133a, miR133b	COL1A1 COL3A1	Amostras cardíacas	Ratos Wistar Treinamento de natação	45
↑ miR-126	SPRED1 PI3KR2	Amostras cardíacas	Ratos Wistar Treinamento de natação	46
↑ miR-21, miR-144, miR-145 ↓ miR-124	PTEN PIK3A TSC2	Amostras cardíacas	Ratos Wistar Treinamento de natação	20
↑ miR-336-5p, miR-130b-5p, let7d-3p, miR-466c-5p, miR-324-3p, miR-146b-5p, miR-132-3p, miR-21-5p, miR-187-3p, miR-29b-5p, miR-324-5p, miR-214-5p, miR-140-5p, miR-152-5p, miR-99b-5p, miR-130a-5p, miR-455-5p, miR-27b-3p, miR-23b-3p, miR-652-5p, miR-199a-3p, miR-223-5p, miR-421-3p, miR-27a-5p, miR-24-5p, miR-34a-3p, miR-140-3p, miR-125b-5p, miR-145a-5p, miR-192-5p, miR-139-5p, miR-199a-5p, miR-674-3p, miR-191-5p, miR-28-3p, miR-195-5p, miR-598, miR-429, miR-224, miR-425, miR-221 ↓ miR-701-5p, miR-220, miR-144-3p, miR-694, miR-485-3p, miR-136-5p, miR-384-3p, miR-376c-3p, miR-208b-3p, miR-411-3p, miR-141-5p, miR-1894-3p, miR-9a, miR-687, miR-451-5p	TNF-α COL1A1 MMP9 PTEN AKT1 AMPK BCL2	Amostras cardíacas	Ratos Wistar Treinamento aeróbico de corrida	22
↑ miR-503, miR-465b-5p, miR-542-3p ↓ miR-652		Amostras cardíacas	Ratos C57Bl6 Treinamento de natação	47
↓ miR-26b, miR-143	IGF1R GATA-4 NFAT1C GSK3B	Amostras cardíacas	Ratos Balb/c Treinamento aeróbico em rodas de metal	48
↑ miR-21, miR-30b ↓ miR-1	BCL-2 p53 PDCD4	Amostras cardíacas	Treinamento de natação	49
↑ miR-23a, miR-27a	PTEN Casp7 FoxO1	Amostras musculoesqueléticas	Exercício de resistência	50
↑ **miR-29c** ↓ **miR-1**	COL1A1 COL3A1	Amostras cardíacas	Treinamento de natação	51
↑ **miR-382**		Amostras de soro, tecido e células	Exercício aeróbico em ratos IR	25
**MicroRNAs**	**Alvos**	**Fonte**	**Tipos de exercícios**	**Referência**
**Estudos clínicos**				
↑ miR-126, miR-133	CPK	Plasma	Espiroergometria limitada a um único sintoma Corrida de maratona Exercício excêntrico de resistência	52
↓ miR-486	PTEN	Soro	Ciclismo sistemático a 70% VO2max	53
↑ miR-1, miR-126, miR-133a, miR-134, miR-146a, miR-208a, miR-499-5p	CPK NT-proBNP hsCRP	Plasma	Corrida de maratona Imediatamente após a corrida	21
↑ miR-1, miR-133a, miR-206, miR-208b, miR-499		Plasma	Corrida de maratona Imediatamente após a corrida	54
↑ miR-1, miR-133a, miR-206		Plasma	Corrida de maratona Imediatamente após a corrida	55
↑ let-7d-3p, let-7f-3p miR-29a-3p, miR-34a-5p, miR-125b-5pmiR-132-3p, miR-143-3p, miR-148a-3p, miR-223-3p, miR-223-5p miR-424-3p, miR-424-5p		Soro	Corrida de maratona Imediatamente após a corrida	56
↑ miR-1, miR-30a, miR-133a ↓ miR-26a, -29b		Plasma	Corrida de maratona Imediatamente após a corrida	57
↑ miR-1, miR-133a, miR-206		Plasma	Corrida de maratona Imediatamente após a corrida	58
↑ miR-1, miR-133a, miR-133b, miR-139-5p, miR-143, miR-145, miR-223, miR-330-3p, miR-338-3p, miR-424 ↓ miR-30b, miR-106a, miR-146, miR-151-3p, miR-151-5p, miR-221, miR-652, let-7i ↑ miR-103, miR-107 ↓ miR-21, miR-25, miR-29b, miR-92a, miR-133a, miR-148a, miR-148b, miR-185, miR-342-3p, miR-766, let-7d		Plasma	Teste cicloergômetro 1-3 h após o exercício Ciclo de resistência sistemática treinamento ergométrico	59
↑ miR-1, miR-133a, miR-133b, miR-206 miR-485-5p, miR-509-5p, miR-517a miR-518f, miR-520f, miR-522, miR-553, miR-888		Plasma	Treinamento intervalado de alta intensidade Imediatamente após	60
↑ miR-181b, miR-214 ↑ miR-1, miR-133a, miR-133b, miR-208b		Plasma	Teste de esteira em aclive (concêntrico) Imediatamente após Teste de esteira em declive (excêntrico) 2-6 h após o exercício	61
↑ miR-149 ↓ miR-146a, miR-221		Soro	Exercício de resistência 3 dias após o exercício	62
↑ miR-1, miR-133a, miR-133b, miR-206, miR-208b, miR-499		Plasma	Treinamento de resistência sistemático 36-72 h após o exercício	63
↑ miR-1, miR-133a, miR-133b, miR-181a ↓ miR-9, miR-23a, miR-23b, miR-31 ↑ miR-1, miR-29b	HDAC4 NRF1	Amostras musculoesqueléticas	Teste cicloergômetro, ciclismo	64
↑ miR-136, miR-200c, miR-376a, miR-377, miR-499b, miR-558 ↓ miR-28, miR-30d, miR-204, miR-330, miR-345, miR-375, miR-449c, miR-483, miR-509, miR-520a, miR-548a, miR-628, miR-653, miR-670, miR-889, miR-1245a, miR-1270, miR-1280, miR-1322, miR-3180		Amostras musculoesqueléticas	Treinamento de resistência	65
↑ miR-451 ↓ miR-26a, miR-29a, miR-378		Amostras musculoesqueléticas	Exercício de resistência	66
↑ miR-125a, miR-145, miR-181b, miR-193a, miR-197, miR-212, miR-223, miR-340, miR-365, miR-485, miR-505, miR-520d, miR-629, miR-638, miR-939, miR-940, miR-1225, miR-1238 ↓ let-7i, miR-16, miR-17, miR-18a, miR-18b, miR-20a, miR-20b, miR-22, miR-93, miR- 96, miR-106a, miR-107, miR-126, miR-130a, miR-130b, miR-151, miR-185, miR-194, miR-363, miR-660		Soro	Exercício em cicloergômetro, ciclismo	67
↑ miR-7, miR-15a, miR-21, miR-26b, miR-132, miR-140, miR-181a, miR-181b, miR-181c, miR-338, miR-363, miR-939, miR-940, miR-1225 ↓ let-7e, miR-23b, miR-31, miR-99a, miR-125a, miR-125b, miR-126, miR-130a, miR-145, miR-151, miR-199a, miR-199b, miR-221, miR-320, miR-451, miR-486, miR-584, miR-652		PBMC	Exercício em cicloergômetro, ciclismo	68
↑ let-7f, miR-21, miR-29c, miR-223 ↓ let-7f, miR-21, miR-29c, miR-223		PBMC	Exercício de corrida	69
↑ miR-7, miR-29a, miR-29b, miR-29c, miR-30e, miR-142, miR-192, miR-338, miR-363, miR-590 ↓ let-7e, miR-126, miR-130a, miR-151, miR-199a, miR-221, miR-223, miR-326, miR-328, miR-652		PBMC	Exercício em cicloergômetro, ciclismo	70
↑ miR-15a, miR-29b, miR-29c, miR-30e, miR-140, miR-324, miR-338, miR-362, miR-532, miR-660 ↓ miR-23b, miR-130a, miR-151, miR-199a, miR-221		Soro	Exercício em cicloergômetro, ciclismo	71
↑ miR-1, miR-486, miR-494		Soro	(Atletas de resistência, corredores, ciclistas e triatletas) Teste de exercício cardiopulmonar	72
↑ miR-21, miR-146a, miR-221, miR-222 ↑ miR-20a, miR-21, miR-146a, miR-221, miR-222		Soro	Treinamento por remada, 5 km, 1-3 h por sessão, 20-24 remadas/min)	73
↑ miR-376a ↓ miR-16, miR-27a, miR-28		Plasma	Treinamento de exercício aeróbico - corrida (4 dias/semana)	74
↑ miR-19a, miR-19b, miR-20a, miR-26b, miR-143, miR-195	p-AKT p-S6K1	Soro	Exercício de resistência	75
↑ miR-222	HIPK1	Plasma	Teste ergométrico em bicicleta	41
↑ miR-221 ↓ miR-208b, miR-221, miR-21, miR-146a, miR-210		Soro	Exercício de basquete	76

Alguns estudos também relataram a importância do TEF na modulação da expressão dos miRNAs em situações patológicas, bem como em diabéticos,^24,25^ na obesidade,^26^ após o infarto do miocárdio^27^ e com insuficiência cardíaca;^22^ entretanto, o papel do TEF na modulação dos miRNAs na DC ainda não foi evidenciado. A literatura apresenta apenas estudos que demonstraram os efeitos benéficos do TEF na DC; porém eles não analisaram o perfil do miRNA.

Realizando apenas TEF aeróbico com intensidade moderada (50 a 70% de frequência cardíaca máxima), três vezes por semana, por 30 minutos, em 12 semanas, obteve-se um aumento significativo na capacidade cardiorrespiratória metabólica máxima (VO2), aumento de tempo de exercício, distância percorrida e melhoria de aspectos emocionais,^28^ e, além disso, em associação com um programa de TR, foram obtidos resultados benéficos.^29^

Outro estudo, com um protocolo de TEF semelhante, também evidenciou uma melhoria da capacidade funcional, com melhoria da fração de ejeção e resistência respiratória, melhoria da pressão diastólica no ventrículo esquerdo, e da qualidade de vida de pacientes chagásicos após 8 meses de treinamento.^30^

Um programa de reabilitação cardíaca composto do mesmo protocolo de TEF mencionado acima, com TR e alongamentos, orientação nutricional adicional e aconselhamento farmacológico para pacientes com DC, demonstrou aumento da capacidade funcional e física, melhorando a qualidade de vida de pacientes chagásicos.^31^

Em outro estudo importante, os pesquisadores realizaram TEF três vezes por semana em pacientes chagásicos. Eles demonstraram que o grupo que realizou exercícios aumentou o consumo de oxigênio de pico durante o exercício e a ventilação máxima por minuto, melhorando a capacidade funcional desses pacientes.^32^

Entretanto, apesar de demonstrar que o TEF tem efeitos benéficos para pacientes com DC, é difícil analisar os efeitos desse tipo de treinamento no nível do tecido, celular e molecular, considerando que esses estudos foram realizados em seres humanos, para quem seriam necessárias biópsias. Portanto, para investigar os possíveis mecanismos associados a esses efeitos benéficos do TEF na DC, alguns estudos foram realizados em modelos experimentais de DC *in vivo*.

Camundongos Balb/c realizaram TEF em uma esteira antes de serem infectados com *T. Cruzi*. Observou-se que o TEF reduziu o pico da parasitemia, concluindo que o TEF pode promover alterações benéficas no sistema imune e obter melhores respostas a infecções.^33^

Em outros estudos, foram relatados os mesmos achados que os de estudos anteriores; entretanto, eles também observaram que ratos que fizeram o treinamento obtiveram maior proteção da atividade metabólica de NADH em neurônios mioentéricos e maior síntese de TNF-α e TGF-β.^34^ Isso contribuiu para a sobrevivência e/ou proteção de 10,3% dos neurônios mioentéricos e sua produção imunorreativa de sintase neuronal de óxido nítrico. O grupo em treinamento, inclusive, obteve maior expressão de TNF-α durante a fase aguda da infecção por *T. Cruzi*, oferecendo benefícios ao sistema imune para preservar os neurônios nitrérgicos.^35^

Nesse contexto, em outro estudo, pesquisadores observaram que o grupo de TEF obteve maior expressão de TNF-α, IFNγ, IL-6 e as quimiocinas MCP-1 e CX3CL1 durante a infecção aguda, além de alcançarem melhor capacidade física, aumento do limiar anaeróbio, aumento da atividade da catalase e do superóxido dismutase, e redução da oxidação lipídica e proteica no tecido cardíaco, demonstrando que o TEF pode ser uma estratégia interessante para aumentar a eficiência de mecanismos antioxidantes endógenos, reduzindo os danos oxidativos nesses animais.^36^

Outro estudo demonstrou que o TEF antes da infecção em ratos Wistar aumentou o tempo até que se atingisse a fadiga e o limiar anaeróbio, reduziu a expressão de TNF-α, CCL-2, MCP-1 e CX3CL1, bem como a oxidação lipídica e proteica, e aumentou a expressão de IL-10, catalase e superóxido dismutase, indicando que o TEF induz um fenótipo protetor, aumentando as defesas do hospedeiro contra o agente parasítico, inclusive atenuando o processo de remodelação patológica associado à miosite musculoesquelética.^37^

Finalmente, em outro estudo, ratos suíços foram infectados pelo *T. Cruzi* após TEF de intensidade moderada em uma esteira, realizado durante 9 semanas. Os pesquisadores identificaram que o TEF conseguiu reduzir a parasitemia latente dos animais infectados submetidos a treinamento, corroborando os achados de estudos anteriores. Eles chegaram a obter menor produção de citocinas pró-inflamatórias (TNF-α, INFγ, IL-12) e proteína quimiotática de monócitos 1 (MCP-1) durante os primeiros dias de infecção.^38^

Portanto, sugere-se que o TEF tenha um potencial terapêutico para a prevenção e o tratamento complementar de DC e CCC pela modulação do sistema imune. Entretanto, estudos clínicos carecem de análises morfométricas, celulares e moleculares especialmente pela análise de miRNAs para melhor entendimento dos efeitos benéficos do TEF na sinalização celular em seres humanos com DC, enquanto estudos pré-clínicos, in vivo, demandam estudos que avaliem os efeitos de TEF com DC e CCC já instaladas e não apenas no estágio de pré-infecção.

## Sobreposições entre miRNAs em DC e TEF

Além disso, realizamos uma análise utilizando o diagrama de Venn, para identificar os miRNAs que foram modulados por TEF, em estudos clínicos e pré-clínicos que podem possivelmente modular miRNAs na DC.

**Figura 1:**
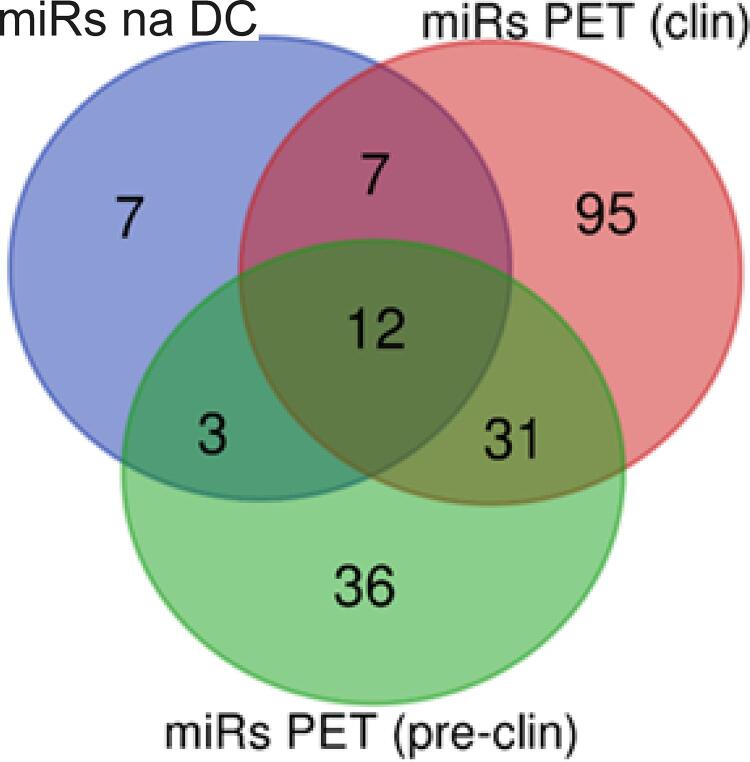
– O diagrama de Venn mostra sobreposições entre miRNAs: miRNAs (miRs) na doença de Chagas (azul), miRs TEF clin: estudos clínicos (rosa), e miRs TEF pre-clin: estudos pré-clínicos (verde).

Houve apenas 7 miRNAs expressos em DC, 95 miRNAs expressos em estudos clínicos com TEF, e 36 miRNAs expressos em estudos pré-clínicos com TEF. É interessante notar que foram identificados 7 miRNAs que tinham modulação tanto na DC quanto em estudos clínicos com TEF, 3 miRNAs comuns modulados na DC e em estudos pré-clínicos com TEF, e, principalmente, 12 miRNAs comuns modulados na CD, estudos clínicos com TEF, e estudos pré-clínicos com TEF (Figura 1). Esses 12 miRNAs são: miR-1, miR-21, miR-26b, miR-29b, miR-133a, miR-133b, miR-139, miR-145, miR-146a, miR-208a, miR-208b, miR-222.

**Figura 2:**
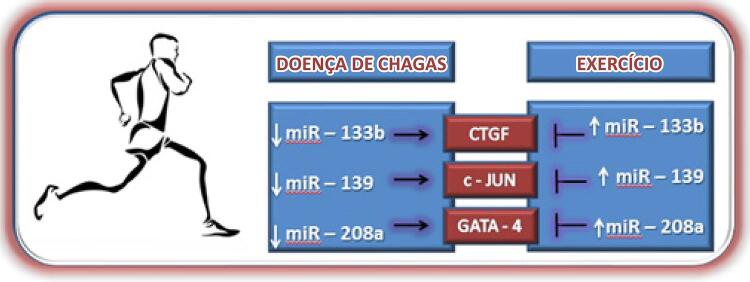
– miRNAs expressos na DC que podem provavelmente ser modulados por TEF

Entretanto, desses 12 miRNAs comuns, apenas miR-133b, miR-139 e miR-208a foram identificados com um padrão de expressão diferente na DC e TEF; todos os 3 miRNAs passam por regulação descendente na DC, e ascendente, em TEF (Figura 2).

O miR-133b controla o fator de crescimento de tecido conjuntivo (CTGF)^77^ e pode suprimir a remodelação cardíaca;^78^ portanto, o TEF pode ser uma alternativa excelente para controlar a remodelação cardíaca, possivelmente pela modulação do miR-133b e a modificação da sinalização celular.

O miR-139 está associado à cardiomiopatia hipertrófica, regulando a expressão do c-Jun, um fator transcricional que liga a região promotora de alguns genes para induzir a hipertrofia cardíaca, e, portanto, a superexpressão desse miRNA reduz a expressão do c-Jun e consequentemente atenua a hipertrofia cardíaca patológica,^79^ que pode ser uma sinalização celular pela qual o TEF suprime a hipertrofia patológica na DC, porque o TEF também aumenta a expressão desse miRNA.^22,59^

Nesse contexto, o miR-208a regula a expressão de alguns fatores transcricionais, tais como GATA-4, que está associado à ativação de genes cardíacos pró-hipertróficos.^80^ Na DC, esse miRNA sofre regulação descendente,^16^ enquanto a TEF pode aumentar sua expressão,^21,42^ demonstrando, portanto, que possivelmente pode ser um mecanismo molecular pelo qual o TEF atenua a hipertrofia cardíaca nessa doença.

## Conclusões

Os miRNAs participam de vários processos na patogênese da DC. Muitas evidências mostram os efeitos benéficos do TEF na DC; entretanto, ainda não há artigos na literatura que demonstrem as alterações nos mecanismos moleculares dos miRNAs que o TEF induz na DC. Dessa forma, são necessários estudos posteriores para elucidar esses mecanismos.
