# Supplementary material for: Treinamento com Exercício Físico e Doença de Chagas: Função Potencial dos MicroRNAs
Source: Arq Bras Cardiol. 2021 Jul 15;117(1):132–41. [Article in Portuguese] doi: 10.36660/abc.20200330 (PMC8294722; doi:10.36660/abc.20200330)
Supplement: Translation

# Translation: Physical Exercise Training and Chagas Disease: Potential Role of MicroRNAs

**Authors:** Alex Cleber Improta-Caria, Roque Aras

**Affiliations:** 1 BrasilPrograma de Pós-Graduação em Medicina e Saúde, Faculdade de Medicina, Universidade Federal da Bahia, Salvador, BA - Brasil; 2 BrasilDepartamento de Educação Física em Cardiologia do Estado da Bahia, Sociedade Brasileira de Cardiologia,Salvador, BA - Brasil

**Keywords:** Exercise, Chagas Disease, MicroRNAs

## Abstract

Chagas disease (CD) is caused by *Trypanosoma Cruzi*. This parasite can infect several organs of the human body, mainly the heart, causing inflammation, fibrosis, arrhythmias, and cardiac remodeling, promoting long-term Chronic Chagas Cardiomyopathy (CCC). However, little scientific evidence has elucidated the molecular mechanisms that govern the pathophysiological processes in this disease. MicroRNAs (miRNAs) are regulators of post-transcriptional gene expression that modulate signaling pathways, participating in pathophysiological mechanisms in CD, but the understanding of miRNAs in this disease is limited. On the other hand, a wide range of scientific evidence shows that physical exercise training (PET) modulates the expression of miRNAs by modifying different signaling pathways in healthy individuals. Some studies also show that PET is beneficial for individuals with CD; however, these did not evaluate the miRNA expressions. Thus, there is no evidence showing the role of PET in the expression of miRNAs in CD. Therefore, this review aimed to identify miRNAs expressed in CD that could potentially be modified by PET.

## Introduction

Chagas Disease (CD) is a complex disease caused by *Trypanosoma Cruzi *(*T. cruzi*), a flagellated protozoan parasite, infection at the intracellular level.^1^ In the acute phase, the *T. cruzi* infection generates great tissue inflammation, and there is an initial response of the innate immune system in an attempt to fight parasitemia.^2^

However, the infection persists and the adaptive immune system activates the T lymphocytes, as well as the auxiliary and cytotoxic T cells, which produce cytokines, such as gamma interferon (IFN-γ), which can in turn lead to intracellular parasitic death by inducing an increase in the reactive oxygen species and nitrogen, which are microbicides. This infection also increases the expression of the tumor necrosis factor (TNF-α) and specific antibodies to combat *T. cruzi*, which control parasitism, with a low-grade infection being established.^3^

Still in the acute phase of the disease, there is an increase in the expression of the vasoactive peptide endothelin-1 (ET-1) and cardiotrophin-1 (CT-1), both inducing cardiac hypertrophy, as well as an increase in the expression of interleukin-1 beta. (IL-1β), inducing an inflammatory and pro-hypertrophic response of the myocardium, which may initiate cardiac hypertrophy even at this stage.^4,5^

Over the years, parasitemia is reduced; however, parasitic antigens persist, generating a diffuse inflammatory infiltrate and myocarditis, with the presence of CD4 + and CD8 + T lymphocytes and macrophages that continue to express TNF-α and IFN-γ.^3^ IFN-γ has an essential function to control and fight against parasites, but it also contributes to cardiac pathogenesis, as it damages the myocardium through several molecular mechanisms generating myocardial dysfunction.^6^

Thus, the disease evolves and passes to the chronic phase, which can be subdivided into two forms: indeterminate and symptomatic. In the indeterminate form, individuals can go for years without manifesting any type of more serious symptom, where there is a balance between parasitemia and the host’s immune system. However, about 30% of these patients develop a symptomatic or determined form, which can trigger dysfunctions in different organs, including the heart, developing Chronic Chagas Cardiomyopathy (CCC) associated with myocarditis and cardiac myofibrillary fibrosis, thereby reducing cardiac electrical conductivity and generating myocardial remodeling.^7^

CCC generates inflammation of the cardiac tissue, causing focal or diffuse myocarditis, hypertrophy, or dilation of the left ventricle and progressive death of some cardiomyocytes, necrosis, and collagen deposit,^8^ thereby increasing the fibrotic tissue, leading to a reduction in its contractile capacity. This outcome is mostly associated with arrhythmias and heart failure,^9^ but microRNAs (miRNAs) may also participate in these mechanisms. In general, the molecular mechanisms that govern these processes are poorly understood.

MiRNAs are small RNAs, with only 18 to 25 nucleotides in length;^10^ non-coding proteins; and regulators of post-transcriptional gene expression with the function of inhibiting or degrading its target genes.^11,12^ It has been shown that several types of physical exercise training (PET) modulate the expression of miRNAs.^13^ Nevertheless, articles that analyze the effects of PET on the expression of miRNAs in CD are still scarce in the literature. Thus, our literature review sought to analyze the miRNAs expressed in CD and to compare this finding with the miRNAs expressed during or after PET.

### Chagas Disease and miRNAs

Few studies in the literature have analyzed the expression profile of miRNAs in CD, either in the acute or in the chronic phase, as well as the signaling pathways that are regulated by miRNAs in this neglected disease. Therefore, this study included all of the studies that evaluated the expression pattern of miRNAs in CD (Table 1).

**Table 1:** – MicroRNAs in Chagas Disease

MicroRNAs	Source	Findings	Reference
↓ miR-1, miR-133a, miR-133b, miR-208a, miR-208b	Heart samples	Association with connective tissue disorders and fibrosis	16
↑ miR-208b	Plasma samples	Association with cardiovascular dysfunction and myocardial hypertrophy	17
↑ miR-20, miR-20b, miR-21, miR-142, miR-146a, miR-146b, miR-155, miR-182, miR-203, miR-222 ↓ miR-139, miR-145, miR-149, miR-322, miR-503,	Heart samples	Association with heart rate-corrected QT (QTc) interval. Ventricular depolarization and repolarization.	14
↑ miR-19a, miR-21, miR-29b, miR-30a, miR-199b	Heart samples and cell model	Association with fibrosis and cardiac remodeling	18
↑ miR-16, miR-26b, miR-190b, miR-3586, let-7f-2 ↓ miR-190b	H9c2 cells, infected with T. Cruzi	Association with cell growth, hypertrophy, and cell survival	19

#### Chagas Disease (acute phase) and miRNAs

During the acute phase of CD, the researchers evaluated the expression of miRNAs at 15, 30, and 45 days post-infection, and identified that miRNAs were differentially expressed during parasitemia and that changes in the QT interval were upregulated: miR-20, miR-20b, miR-21, miR-142, miR-146a, miR-146b, miR-155, miR-182, miR-203, and miR-222, and downregulated: miR-139, miR-145, miR-149, miR-322, and miR-503.^14^

Another study performed an *in silico* analysis to identify the differential expression of miRNAs and their target genes in several biological processes during the acute phase of *T. Cruzi* infection, demonstrating that some miRNAs may be associated with the pathological process, such as miR-238-3p, miR-149-5p, miR-143-3p, miR-145-5p, and miR-486-5p. Other miRNAs may be associated with cardiovascular immunity and function, for example: miR-10a-5p, miR-16-5p, miR-30c-5p, miR-34a-5p, miR-138-5p, miR-146a-5p, miR-149, miR-191-5p, miR-204-5p, miR-320b and miR-653-3p, as well as miRNAs related to the tissue fibrosis process: miR-34a-5p, miR-142-3p, miR-200b-3p, and 203a-3p.^15^

#### Chagas Disease (chronic phase) and miRNAs

The expression of miRNAs from the cardiac tissue of patients with CCC after heart transplantation was analyzed and compared with the expression of miRNAs from the cardiac tissue of healthy donor individuals. Of all miRNAs analyzed, five miRNAs had their expression reduced (miR-1, miR-133a, miR-133b, miR-208a, and miR-208b) in patients with CCC when compared to the control group.^16^ By contrast, the circulating miR-208a in a plasma sample was overexpressed in patients with CD; however, these were in the undetermined chronic phase.^17^

The overexpression of MiR-19a, miR-21, and miR-29b has been described in patients with CCC when compared to healthy individuals. In fact, in the histological analysis of the cardiac tissue of patients in the final stage of CCC, it was identified that, in addition to the miRNAs mentioned above, the miR-30a and miR-199b are also overexpressed in the CD.^18^

These studies demonstrate that many miRNAs participate in several processes in the CD both in the acute and chronic phase; however, further studies are needed to elucidate the role of these miRNAs and the signaling pathways they are regulating in the CD, including the importance of therapies or treatments that can modulate the pattern of expression shown in the disease.

## Chagas Disease and Physical Exercise Training: miRNAs as potential modulators

Several types of PET have been described as modulators of the expression of many miRNAs,^13^ in experimental and clinical studies, such as swimming PET,^20^ marathon,^21^ running on a treadmill,^22^ and resistance training (RT)^23^ (Table 2).

**Table 2:** – MicroRNAs in Physical Exercise Training (pre-clinical and clinical studies)

MicroRNAs	Target	Source	Types of Exercises	Reference
**In vivo experimental models**				
↑** miR-27a, miR-155** ↓** miR-143**	ACE, AT1R	Heart samples	Wistar-Kyoto rats Exercise training on treadmill	39
↑ **miR-17-3p**	TIMP-3 PTEN	Heart samples	C57Bl/6 mice Ramp swimming training model Voluntary wheel training	40
↑ miR-222	HIPK1	Heart samples	Ramp swimming model Voluntary wheel training	41
↑ miR-19b, miR-30e, miR-133b, miR-208a ↓ miR-99b, miR-100, miR-191a, miR-22, miR-181a	IGF-1 PI3K/AKT/mTOR MAPK	Heart samples Plasma	Wistar albino rats Swimming training	42
↑ miR-29a, miR-101a	TG-β fos COL1A1	Heart samples	Intermittent run exercise	43
↑ miR-27a, miR-27b ↓ miR-143	ACE ACE2	Heart samples	Wistar rats Swimming training	44
↑ miR-126	PI3KR2	Heart samples Plasma	Zucker rats Swimming training	26
↓ miR-214	SERCA2A	Heart samples	Wistar rats Resistance training	23
↑ miR-1 ↓ miR-214	NCX SERCA2A	Heart samples	Wistar rats Swimming training	27
↑ miR-29c ↓ miR-1, miR-133a, miR133b	COL1A1 COL3A1	Heart samples	Wistar rats Swimming training	45
↑ miR-126	SPRED1 PI3KR2	Heart samples	Wistar rats Swimming training	46
↑ miR-21, miR-144, miR-145 ↓ miR-124	PTEN PIK3A TSC2	Heart samples	Wistar rats Swimming training	20
↑ miR-336-5p, miR-130b-5p, let7d-3p, miR-466c-5p, miR-324-3p, miR-146b-5p, miR-132-3p, miR-21-5p, miR-187-3p, miR-29b-5p, miR-324-5p, miR-214-5p, miR-140-5p, miR-152-5p, miR-99b-5p, miR-130a-5p, miR-455-5p, miR-27b-3p, miR-23b-3p, miR-652-5p, miR-199a-3p, miR-223-5p, miR-421-3p, miR-27a-5p, miR-24-5p, miR-34a-3p, miR-140-3p, miR-125b-5p, miR-145a-5p, miR-192-5p, miR-139-5p, miR-199a-5p, miR-674-3p, miR-191-5p, miR-28-3p, miR-195-5p, miR-598, miR-429, miR-224, miR-425, miR-221 ↓ miR-701-5p, miR-220, miR-144-3p, miR-694, miR-485-3p, miR-136-5p, miR-384-3p, miR-376c-3p, miR-208b-3p, miR-411-3p, miR-141-5p, miR-1894-3p, miR-9a, miR-687, miR-451-5p	TNF-α COL1A1 MMP9 PTEN AKT1 AMPK BCL2	Heart samples	Wistar rats Aerobic run training	22
↑ miR-503, miR-465b-5p, miR-542-3p ↓ miR-652		Heart samples	C57Bl6 mice Swimming training	47
↓ miR-26b, miR-143	IGF1R GATA-4 NFAT1C GSK3B	Heart samples	Balb/c mice Aerobic metal wheels training	48
↑ miR-21, miR-30b ↓ miR-1	BCL-2 p53 PDCD4	Heart samples	Swimming training	49
↑ miR-23a, miR-27a	PTEN Casp7 FoxO1	Skeletal muscle samples	Resistance exercise	50
↑ **miR-29c** ↓ **miR-1**	COL1A1 COL3A1	Heart samples	Swimming training	51
↑ **miR-382**		Serum, tissues, and cell samples	IR mice Aerobic exercise	25
**MicroRNAs**	**Targets**	**Source**	**Types of Exercises**	**Reference**
**Clinical studies**				
↑ miR-126, miR-133	CPK	Plasma	Single symptom-limited spiroergometry test Marathon run Eccentric resistance exercise	52
↓ miR-486	PTEN	Serum	Systematic-cycling at 70% VO2max	53
↑ miR-1, miR-126, miR-133a, miR-134, miR-146a, miR-208a, miR-499-5p	CPK NT-proBNP hsCRP	Plasma	Marathon run Immediately after run	21
↑ miR-1, miR-133a, miR-206, miR-208b, miR-499		Plasma	Marathon run Immediately after run	54
↑ miR-1, miR-133a, miR-206		Plasma	Marathon run Immediately after run	55
↑ let-7d-3p, let-7f-3p miR-29a-3p, miR-34a-5p, miR-125b-5pmiR-132-3p, miR-143-3p, miR-148a-3p, miR-223-3p, miR-223-5p miR-424-3p, miR-424-5p		Serum	Marathon run Immediately after run	56
↑ miR-1, miR-30a, miR-133a ↓ miR-26a, -29b		Plasma	Marathon run Immediately after run	57
↑ miR-1, miR-133a, miR-206		Plasma	Marathon run Immediately after run	58
↑ miR-1, miR-133a, miR-133b, miR-139-5p, miR-143, miR-145, miR-223, miR-330-3p, miR-338-3p, miR-424 ↓ miR-30b, miR-106a, miR-146, miR-151-3p, miR-151-5p, miR-221, miR-652, let-7i ↑ miR-103, miR-107 ↓ miR-21, miR-25, miR-29b, miR-92a, miR-133a, miR-148a, miR-148b, miR-185, miR-342-3p, miR-766, let-7d		Plasma	Cycle ergometry test 1-3 hs after exercise Systematic endurance cycle ergometry training	59
↑ miR-1, miR-133a, miR-133b, miR-206 miR-485-5p, miR-509-5p, miR-517a miR-518f, miR-520f, miR-522, miR-553, miR-888		Plasma	High intensity interval exercise Immediately after	60
↑ miR-181b, miR-214 ↑ miR-1, miR-133a, miR-133b, miR-208b		Plasma	Uphill treadmill test (concentric) Immediately after Downhill treadmill test (eccentric) 2-6 hs after exercise	61
↑ miR-149 ↓ miR-146a, miR-221		Serum	Resistance exercise 3 days after exercise	62
↑ miR-1, miR-133a, miR-133b, miR-206, miR-208b, miR-499		Plasma	Systematic resistance training 36-72 hs after training	63
↑ miR-1, miR-133a, miR-133b, miR-181a ↓ miR-9, miR-23a, miR-23b, miR-31 ↑ miR-1, miR-29b	HDAC4 NRF1	Skeletal muscle samples	Cycle ergometer, Cycling	64
↑ miR-136, miR-200c, miR-376a, miR-377, miR-499b, miR-558 ↓ miR-28, miR-30d, miR-204, miR-330, miR-345, miR-375, miR-449c, miR-483, miR-509, miR-520a, miR-548a, miR-628, miR-653, miR-670, miR-889, miR-1245a, miR-1270, miR-1280, miR-1322, miR-3180		Skeletal muscle samples	Resistance training	65
↑ miR-451 ↓ miR-26a, miR-29a, miR-378		Skeletal muscle samples	Resistance exercise	66
↑ miR-125a, miR-145, miR-181b, miR-193a, miR-197, miR-212, miR-223, miR-340, miR-365, miR-485, miR-505, miR-520d, miR-629, miR-638, miR-939, miR-940, miR-1225, miR-1238 ↓ let-7i, miR-16, miR-17, miR-18a, miR-18b, miR-20a, miR-20b, miR-22, miR-93, miR- 96, miR-106a, miR-107, miR-126, miR-130a, miR-130b, miR-151, miR-185, miR-194, miR-363, miR-660		Serum	Cycle ergometer exercise	67
↑ miR-7, miR-15a, miR-21, miR-26b, miR-132, miR-140, miR-181a, miR-181b, miR-181c, miR-338, miR-363, miR-939, miR-940, miR-1225 ↓ let-7e, miR-23b, miR-31, miR-99a, miR-125a, miR-125b, miR-126, miR-130a, miR-145, miR-151, miR-199a, miR-199b, miR-221, miR-320, miR-451, miR-486, miR-584, miR-652		PBMC	Cycle ergometer exercise	68
↑ let-7f, miR-21, miR-29c, miR-223 ↓ let-7f, miR-21, miR-29c, miR-223		PBMC	Running exercise	69
↑ miR-7, miR-29a, miR-29b, miR-29c, miR-30e, miR-142, miR-192, miR-338, miR-363, miR-590 ↓ let-7e, miR-126, miR-130a, miR-151, miR-199a, miR-221, miR-223, miR-326, miR-328, miR-652		PBMC	Cycle ergometer exercise	70
↑ miR-15a, miR-29b, miR-29c, miR-30e, miR-140, miR-324, miR-338, miR-362, miR-532, miR-660 ↓ miR-23b, miR-130a, miR-151, miR-199a, miR-221		Serum	Cycle ergometer exercise	71
↑ miR-1, miR-486, miR-494		Serum	(Endurance athletes, runners, cyclists, and triathletes) Cardiopulmonary exercise test	72
↑ miR-21, miR-146a, miR-221, miR-222 ↑ miR-20a, miR-21, miR-146a, miR-221, miR-222		Serum	Rowing training, 5Km, 1-3 h per session, 20-24 strokes/min)	73
↑ miR-376a ↓ miR-16, miR-27a, miR-28		Plasma	Aerobic run exercise training (4 days/week)	74
↑ miR-19a, miR-19b, miR-20a, miR-26b, miR-143, miR-195	p-AKT p-S6K1	Serum	Resistance exercise	75
↑ miR-222	HIPK1	Plasma	Bicycle Ergometry Test	41
↑ miR-221 ↓ miR-208b, miR-221, miR-21, miR-146a, miR-210		Serum	Basketball Exercise	76

Some studies have also reported the importance of PET modulating the expression of miRNAs in pathological situations, as well as in diabetics,^24,25^ in obesity,^26^ after myocardial infarction,^27^ and with heart failure;^22^ however, the role of PET-modulating miRNAs in CD has not yet been illustrated. The literature presents only studies that have shown beneficial effects of PET on CD, but they did not analyze the miRNA profile.

Performing only aerobic PET with moderate intensity (50% to 70% of maximum heart rate), three days a week, for 30 minutes, in 12 weeks, obtained a significant increase in maximum cardiorespiratory and metabolic capacity (VO2), increased time in exercise, distance covered, and improvement in emotional aspects,^28^ as well as association with an RT program, obtained similar beneficial results.^29^

In another study, with a similar PET protocol, an improvement in functional capacity was also evidenced, with an improvement in ejection fraction and respiratory strength, improvement in diastolic pressure in the left ventricle and in the quality of life of Chagas patients after 8 months of training.^30^

A cardiac rehabilitation program consisting of the same PET protocol mentioned above, with RT and stretches, adding nutritional guidance and pharmacological counseling for patients with CD, demonstrated an increase in the physical and functional capacity, improving the quality of life of Chagas patients.^31^

In another important study, researchers performed PET three times a week for six months on Chagas patients. They demonstrated that the exercise group increased peak exercise oxygen consumption and maximum minute ventilation, improving the functional capacity of these patients.^32^

However, even demonstrating that PET has beneficial effects for patients with CD, it is difficult to analyze the effects of this type of training at the tissue, cell, and molecular levels, given that these studies were performed in humans, where biopsies would be necessary. Therefore, to investigate the possible mechanisms associated with these beneficial effects of PET on CD, some studies have been carried out on experimental models of CD *in vivo*.

Balb/c mice performed PET on a treadmill before being infected by *T. Cruzi*. It was observed that PET reduced the peak of parasitemia, concluding that PET can promote beneficial changes in the immune system and obtain better responses to infections.^33^

In other studies, the same finding as in the previous study was reported; however, they also observed that trained mice obtained greater protection from the metabolic activity of NADH in myenteric neurons and greater synthesis of TNF-α and TGF-β.^34^ This contributed to the survival and/or protection of 10.3% of myenteric neurons and their immunoreactive production of nitric oxide neuronal synthase, in fact, the trained group obtained a greater expression of TNF-α during the acute phase of *T. Cruzi* infection, providing benefits to the host and improving the immune system to preserve nitrergic neurons.^35^

In this context, in another study, researchers observed that the PET group obtained a greater expression of TNF-α, IFNγ, IL-6, and chemokines MCP-1 and CX3CL1 during acute infection, and also obtained better physical capacity, increased anaerobic threshold, increased activity of catalase and superoxide dismutase and reduced lipid and protein oxidation in cardiac tissue, demonstrating that PET can be an interesting strategy to increase the efficiency of endogenous antioxidant mechanisms, reducing oxidative damage in these animals.^36^

Another study showed that PET before infection in Wistar rats, increased the time to reach fatigue and anaerobic threshold, reduced the expression of TNF-α, CCL-2, MCP-1, and CX3CL1, as well as lipid and protein oxidation, and increased the expression of IL-10, catalase, and superoxide dismutase, indicating that PET induces a protective phenotype, increasing the host’s defenses against the parasitic agent, including the attenuation of the pathological remodeling process associated with musculoskeletal myositis.^37^

Finally, in another study, Swiss mice were infected by *T. Cruzi* after PET with moderate intensity on a treadmill, being carried out for 9 weeks. Researchers identified that PET was able to reduce the latent parasitemia of the infected animals they trained, corroborating with previous studies, and even obtained less production of pro-inflammatory cytokines (TNF-α, INFγ, IL-12) and type-1 monocyte chemotactic protein (MCP-1) during the first days of infection.^38^

Thus, it is suggested that PET has a therapeutic potential for the prevention and complementary treatment of CD and CCC through the modulation of the immune system. However, clinical studies lack morphometric, cellular, and molecular analyzes, mainly through the analysis of miRNAs for a better understanding of the beneficial effects of PET on signaling pathways in humans with CD, while preclinical studies, *in vivo*, need studies that evaluate the effects of PET with CD and CCC already installed and not only in the pre-infection stage.

## Overlaps between miRNAs in CD and PET

Additionally, this study also performed an analysis using the Venn diagram to identify miRNAs that were modulated by PET in both clinical and pre-clinical studies that can possibly modulate miRNAs in CD.

There were only 7 miRNAs expressed in CD, 95 miRNAs expressed in PET clinical studies, and 36 miRNAs expressed in PET pre-clinical studies. Interestingly, the present study identified 7 miRNAs that had modulations in both CD and PET clinical studies, 3 common miRNAs modulated in CD and PET pre-clinical studies and, mainly, 12 common miRNAs modulated in CD, PET clinical studies, and PET pre-clinical studies (Figure 1). These 12 miRNAs are: miR-1, miR-21, miR-26b, miR-29b, miR-133a, miR-133b, miR-139, miR-145, miR-146a, miR-208a, miR-208b, and miR-222.

**Figure 1:** – Venn diagram shows overlaps between miRNAs: miRNAs (miRs) in Chagas Disease (blue), miRs PET clin: clinical studies (pink) and miRs PET pre-clin: pre-clinical studies (green).

Nevertheless, of these 12 common miRNAs, only miR-133b, miR-139, and miR-208a were identified with a different expression pattern in CD and PET; all 3 miRNAs are downregulated in CD and upregulated in PET (Figure 2).

**Figure 2:** – miRNAs expressed in CD that can likely be modulated by PET.

MiR-133b controls the connective tissue growth factor (CTGF)^77^ and can suppress cardiac remodeling;^78^ therefore, PET can be an excellent alternative to control cardiac remodeling, possibly through the modulation of miR-133b and the modification of some signaling pathways.

MiR-139 is associated with hypertrophic cardiomyopathy, regulating the expression of c-Jun, a transcriptional factor that binds in the promoter region of some genes to induce cardiac hypertrophy; thus, the overexpression of this miRNA reduces the expression of c-Jun, and consequently attenuates the pathological cardiac hypertrophy,^79^ which may be a signaling pathway by which PET suppresses the pathological hypertrophy in CD, since PET also increases the expression of this miRNA.^22,59^

In this context, miR-208a regulates the expression of some transcriptional factors, such as GATA-4, which is associated with the activation of pro-hypertrophic cardiac genes.^80^ In CD, this miRNA is downregulated,^16^ while PET can increase its expression,^21,42^ thus demonstrating that it may possibly be a molecular mechanism by which PET attenuates cardiac hypertrophy in this disease.

## Conclusions

miRNAs participate in several processes in the pathogenesis of CD. Much evidence shows the beneficial effects of PET on CD; however, there still are no articles in the literature that demonstrate the changes in the molecular mechanisms of miRNAs that PET induces in CD. Therefore, further studies are necessary to elucidate these mechanisms.
